# Evolutionary radiation and microbial community dynamics shape the thermal tolerance of Fungiidae in the southern South China Sea

**DOI:** 10.1128/spectrum.02436-23

**Published:** 2024-01-04

**Authors:** Yuxin Wei, Biao Chen, Kefu Yu, Zhiheng Liao, Xiaopeng Yu, Zhenjun Qin, Zeming Bao, Lijia Xu, Yongzhi Wang

**Affiliations:** 1 Guangxi Laboratory on the Study of Coral Reefs in the South China Sea, Coral Reef Research Center of China, School of Marine Sciences, Guangxi University, Nanning, China; 2 Southern Marine Science and Engineering Guangdong Laboratory, Guangzhou, China; 3 Key Laboratory of Environmental Change and Resource Use in Beibu Gulf, Ministry of Education, Nanning Normal University, Nanning, China; 4 South China Institute of Environmental Sciences, MEE, Guangzhou, China; College of Life Sciences, Nanchang University, Nanchang, Jiangxi, China

**Keywords:** Fungiidae, evolutionary radiation, microbial community dynamics, thermal tolerance

## Abstract

Fungiidae have shown increased thermal adaptability in coral reef ecosystems under global warming. This study analyzes the evolutionary divergence and microbial communities of Fungiidae in the Sanjiao Reef of the southern South China Sea and explores the impact of coral evolution radiation and microbial dynamics on the heat tolerance of Fungiidae. The results found that *Cycloseris* was an ancient branch of Fungiidae, dating back approximately 147.8953 Mya, and Fungiidae differentiated into two ancestral clades (clades I and II) before 107.0312 Ma. Fungiidae exhibited specific symbioses with the *Cladocopium* C27 sub-clade. Notably, the *Cladocopium* C1 sub-clade has a high relative abundance in clade I, whereas the heat-tolerant *Cladocopium* C40 and C3u sub-clades subdominante in clade II. Regarding bacterial communities, *Cycloseris costulata*, the earliest divergent species, had higher bacterial β-diversity, while the latest divergent species, *Lithophyllon scabra*, displayed lower bacterial α-diversity and higher community stability. Beneficial bacteria dominante Fungiidae’s bacterial community (54%). The co-occurrence network revealed that microbial networks in clade II exhibited lower complexity and greater resilience than those in clade I. Our study highlights that host evolutionary radiation and microbial communities shaped Fungiidae’s thermal tolerance. The variability in subdominant Symbiodiniaceae populations may contribute to interspecific differences in thermal tolerance along the evolutionary branches of Fungiidae. The presence of abundant beneficial bacteria may further enhance the thermal ability of the Fungiidae. Furthermore, the later divergent species of Fungiidae have stronger heat tolerance, possibly driven by the increased regulation ability of the host on the bacterial community, greater microbial community stability, and interaction network resistance.

**IMPORTANCE:**

Coral reefs are facing significant threats due to global warming. The heat tolerance of coral holobionts depends on both the coral host and its microbiome. However, the association between coral evolutionary radiation and interspecific differences in microbial communities remains unclear. In this study, we investigated the role of evolutionary radiation and microbial community dynamics in shaping the thermal acclimation potential of Fungiidae in the Sanjiao Reef of the southern South China Sea. The study’s results suggest that evolutionary radiation enhances the thermal tolerance of Fungiidae. Fungiidae species that have diverged more recently have exhibited a higher presence of heat-tolerant Symbiodiniaceae taxa, more stable bacterial communities, and a robust and resilient microbial interaction network, improving the thermal adaptability of Fungiidae. In summary, this study provides new insights into the thermal adaptation patterns of corals under global warming conditions.

## INTRODUCTION

Although coral reefs are distributed in nutrient-deficient tropical waters (1), they have immense biodiversity and uniqueness. They play important roles in maintaining biodiversity, protecting the marine environment, providing fishery resources, preventing coastal erosion, and supporting leisure tourism and related industries (2–5). However, the coral reef ecosystem is threatened by global warming, resulting in a significant decline in living coral coverage (approximately 50%‒80%) globally since the 1970s (6–9). According to geological records of the South China Sea (SCS), coral reefs developed a similar biodiversity composition and ecosystem scale around 24 Mya (10). Extreme heat events throughout history have not completely destroyed coral reefs. Historically, the fossil records of scleractinian corals date back to the Middle Triassic Period, approximately 240 Mya. These records indicate that scleractinian corals have endured extreme heat events throughout their long evolutionary history, suggesting their remarkable ability to adapt to such conditions (11). Previous studies have shown that corals have developed various morphological types to cope with high-temperature environments (12–14). Measurement of coral tissue thickness in the SCS showed that *Galaxea*, *Favia*, *Platygyra*, and *Porites* had thicker tissues, which enabled corals to have a higher energy reserve and photoprotection capacity, resulting in a higher tolerance to thermal stress (15). It is worth noting that the adaptation and response of corals to the thermal environment are determined by the coral itself and the related microbiome (16–18). Coral holobionts are composed of coral hosts and microbiomes. Members of the microbiome mainly include endosymbiotic Symbiodiniaceae, bacteria, fungi, viruses, and archaea (19). Many studies have suggested that the microbiome is dynamic and diverse, evolves with the coral host, and contributes to the thermal resilience of the coral holobiont (19–22). However, the relationship between the evolutionary radiation of scleractinian corals and the thermal adaptation potential of coral holobionts remains unclear.

During the Triassic period, Scleractinia experienced rapid expansion and adaptive radiation in shallow marine environments, establishing a symbiotic relationship with photosynthetic Symbiotiniaceae (23–25). This symbiotic relationship forms the foundation for corals to survive and dominate in nutrient-deficient waters (26, 27). Based on recent comprehensive studies on the molecular taxonomy, morphology, physiology, and ecology, researchers have identified seven known genera and two undetermined genera within the Symbiotiniaceae family (28). The long-term historical evolution process has created different genera with different physiological characteristics and heat stress adaptation potential (29–31). For instance, compared to *Cladocopium*, corals associated with *Durusdinium* have higher photochemical efficiency of photosystem II and higher ratios of maximum net photosynthesis to respiration. These characteristics enhance corals’ adaptability to thermal stress (32). Compared to the C1 and B1 sub-clades, the A1 sub-clade shows a stronger tolerance to high-temperature stress (33). Additionally, the relative abundance of heat-resistant Symbiodiniaceae types (D1, C15, C3u) increases with decreasing latitudinal gradients, suggesting their role in acclimatizing low-latitude corals to the thermal environment (34). Therefore, by establishing symbiotic relationships with different types of Symbiodiniaceae, corals can acquire diverse adaptations to high-temperature environments. Among the members of the coral holobiont, bacteria have been extensively studied and can be divided into beneficial and pathogenic bacteria based on their functions (18, 35). According to research (36), beneficial microorganisms play a crucial role in the growth and development of corals. These bacteria are involved in activities such as antibiotic secretion, energy cycling, and nutrient metabolism (37–39), and they are closely related to the thermal adaptability of corals (36). Under extremely high-temperature conditions, the abundance of nitrogen-fixing *Cyanobacteria* significantly increased (40). This increase improved the heat tolerance threshold and bleaching recovery rate of corals *via* available nitrogen fixation and transport (41). However, heat stress may increase the relative abundance of pathogenic bacteria and the susceptibility of corals to bleaching (18, 42). *Vibrio shiloi* is a typical pathogen whose relative abundance increases significantly under heat-stress conditions (43, 44), causing an elevated expression of genes associated with virulence factors in corals (18, 45). Notably, the dynamics of coral bacterial communities are closely linked to the maturation of the coral immune system (18). Previous studies have shown that corals have developed a mature and robust immune system through evolutionary divergence to maintain the homeostasis of symbiotic relationships and enhance their thermal adaptation (46, 47). Since the 1980s, many bleaching events have been the result of the disruption of coral immune systems by extreme heat (46). Heat stress can damage the immune systems of corals and deprive them of their ability to regulate bacterial communities, resulting in a significant increase in coral bacterial diversity and community heterogeneity, which is consistent with the Anna Karenina principle (48, 49). For example, when corals are exposed to high temperatures, the relative abundance of beneficial bacteria (e.g., *Endozoicomonas*) in the coral bacterial community decreases substantially. In contrast, the relative abundance of pathogenic bacteria (e.g., Vibrionaceae, Alphaproteobacteria, Rhizobiales, Rhodobacteriales, and Caulobacteriales) increases dramatically (43, 44, 50). This ultimately leads to an increase in the diversity of coral-related microorganisms (51). In addition, in the study of the coral bacterial communities of Hardy Reef (19°44′33″ S, 149°10′57″ E), *Acropora millepora* with the white syndrome was found to have a more discrete bacterial community structure than healthy corals (52). It follows that evolutionary radiation in corals may promote the maturation of their immune systems, and the diversity and heterogeneity of coral-related bacterial communities can serve as markers of coral immune system maturity. Furthermore, a complex microbial network exists within coral microbiomes, where microorganisms engage in direct or indirect associations through processes such as competition, facilitation, and inhibition (53–55) to adapt to disturbances in the thermal environment (56). In conclusion, the variability in tolerance among different coral hosts toward high-temperature stress may be closely linked to their evolutionary radiation, microbial communities, and microbial interactions.

Fungiidae, also known as mushroom corals, were established by Dana in 1846 (57). According to Wells, Fungiidae originated from the extinct Synastridae (Hexacorallia) in the mid-Cretaceous period (57, 58). Fungiidae exhibit a wide range of evolutionary divergence and rich species diversity, and the latest morphological and molecular taxonomy studies have identified 17 genera and approximately 55 species within this family (59). Previous studies have found that Fungiidae are highly adapted to thermal stresses. Baria et al. reported larval survival rates of up to 69%–80% for *Fungia fungites* and 72%–80% for *Lithophyllon repanda* in environments above ambient temperature (2–4°C). These rates were significantly higher than those of *Favia fragum* (18%), which was reported to be highly tolerant to thermal stress (60, 61). Additionally, an increase in sea surface temperature (SST) triggered by global warming has caused massive coral bleaching events since the 1980s, including three extensive coral bleaching events in 1998, 2010, and 2015–2017 (62). In the third global thermal coral bleaching event, abnormally high temperatures affected approximately 75% of the world’s coral reefs quickly and directly destroyed over 1,000 km² of coral habitats (4, 62). In the Australian Great Barrier Reef, 91.1% of the coral reefs were damaged during this catastrophic event. The proportion of coral reefs with >60% bleaching was more than four times higher than that in 1998 or 2002 (63). Additionally, the rapidly growing *Acropora* was nearly extinct (64). However, Hughes et al. showed that Fungiidae have lower levels of bleaching and higher survival rates during this serious bleaching event (>80% bleaching) than other coral species (63). In addition, lagoons are usually considered unfavorable environments for coral growth because of their weak seawater connectivity, weak hydrodynamics, and high SST (65, 66). However, Fungiidae can adapt well to the more extreme lagoon environment in the SCS and even occur in aggregates; similarly, during the SST increase on Parris Island in 1983, Fungiidae living in the lagoon and inner reef flat hardly experienced bleaching (67). The above evidence indicates that Fungiidae have strong heat tolerance and are the winners of coral bleaching events. However, the evolutionary divergence of Fungiidae remains unclear, as do the interspecific differences in microbial communities and their association with the evolutionary radiation of Fungiidae. This contributed to our inability to determine whether the strong thermal tolerance of Fungiidae is influenced by host evolution and microbial community dynamics.

**FIG 1:**
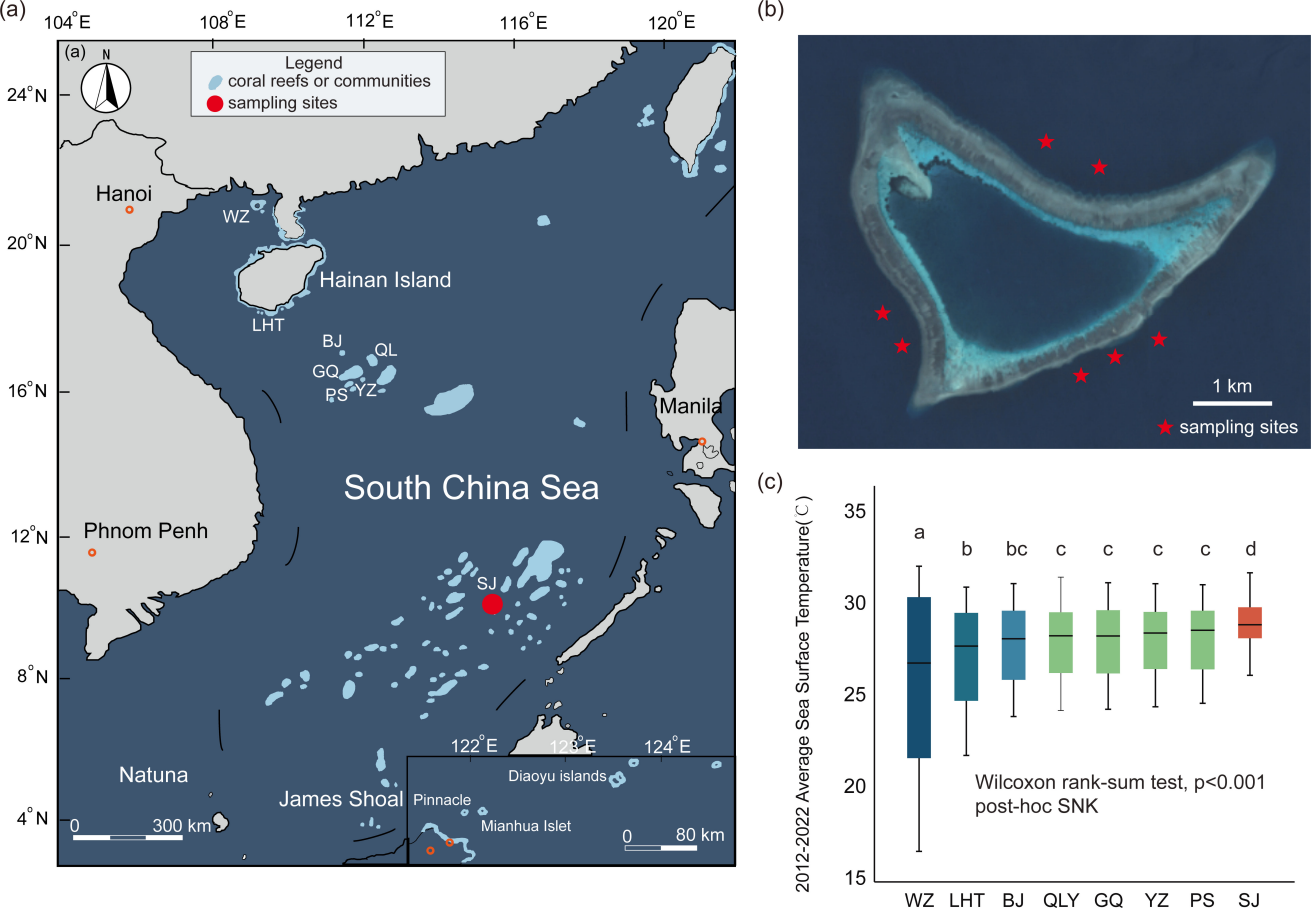
Study areas and sampling sites. (**a**) The blue areas represent the distribution of coral reefs or communities in the SCS. The red circle represents the SJR. (**b**) SJR, the pentagram shapes denote the sampling sites of Fungiidae. (**c**) Spatial differences in monthly SST in the SCS from 2012 to 2022. Weizhou Island (WZ), Luhuitou (LHT), Beijiao (BJ), Qilianyu (QLY), Ganquan Island (GQ), Yuzhuo Reef (YZ), Panshi (PS), Sanjiao Reef (SJ); Wilcoxon rank-sum test.

The SCS is located in the northwestern part of the Coral Triangle and contains abundant coral reef resources. Among them, the Nansha Islands stand out with a remarkable recorded count of approximately 16 families, 73 genera, and 386 species of scleractinian corals (68), which play an important role in supporting the world’s marine biodiversity and other marine resources (69, 70). The Sanjiao Reef (SJR) on the Nansha Islands is located in the southern part of the SCS and is a typical tropical coral reef near the equator (71). In the context of the intensification of global warming, SJR is experiencing a serious threat of thermal bleaching. The result of the monthly average SST (2012–2022; Table S1) analysis showed that the SST of SJR (29.3 ± 1.3°C) was higher than that of the Xisha Islands (e.g., Yuzhuo Reef: 28.1 ± 1.8°C; Fig. 1c). Li et al. analyzed the density of Symbiotiniaceae and satellite remote sensing data, indicating that SJR and Meiji Reef in the Nansha Islands experienced abnormally high temperatures exceeding 30.8°C for four months in 2007. This temperature was higher than the global average SST of coral thermal bleaching of about 0.5°C in 1998, causing severe coral bleaching and mortality (61). Interestingly, Fungiidae living in SJR at high temperatures throughout the year did not die during the previous bleaching events. Therefore, SJR is a natural laboratory for assessing the relationship between the high-temperature acclimation of Fungiidae, host evolution, and microbial community dynamics.

In this study, 28 Fungiidae samples were collected from SJR in the SCS. Based on Sanger sequencing of the COI gene and generation sequencing of the ITS2 and 16S rRNA genes, we analyzed the relationship between evolutionary radiation and the microbial community dynamics of Fungiidae. We explored the driving effects of coral species evolutionary divergence and interspecific differences in microbial community dynamics on the thermal tolerance of Fungiidae. The results of this study provide new insights into the response mechanisms and adaptation patterns of corals to global warming.

## RESULTS

### Evolutionary divergence time

**FIG 2:**
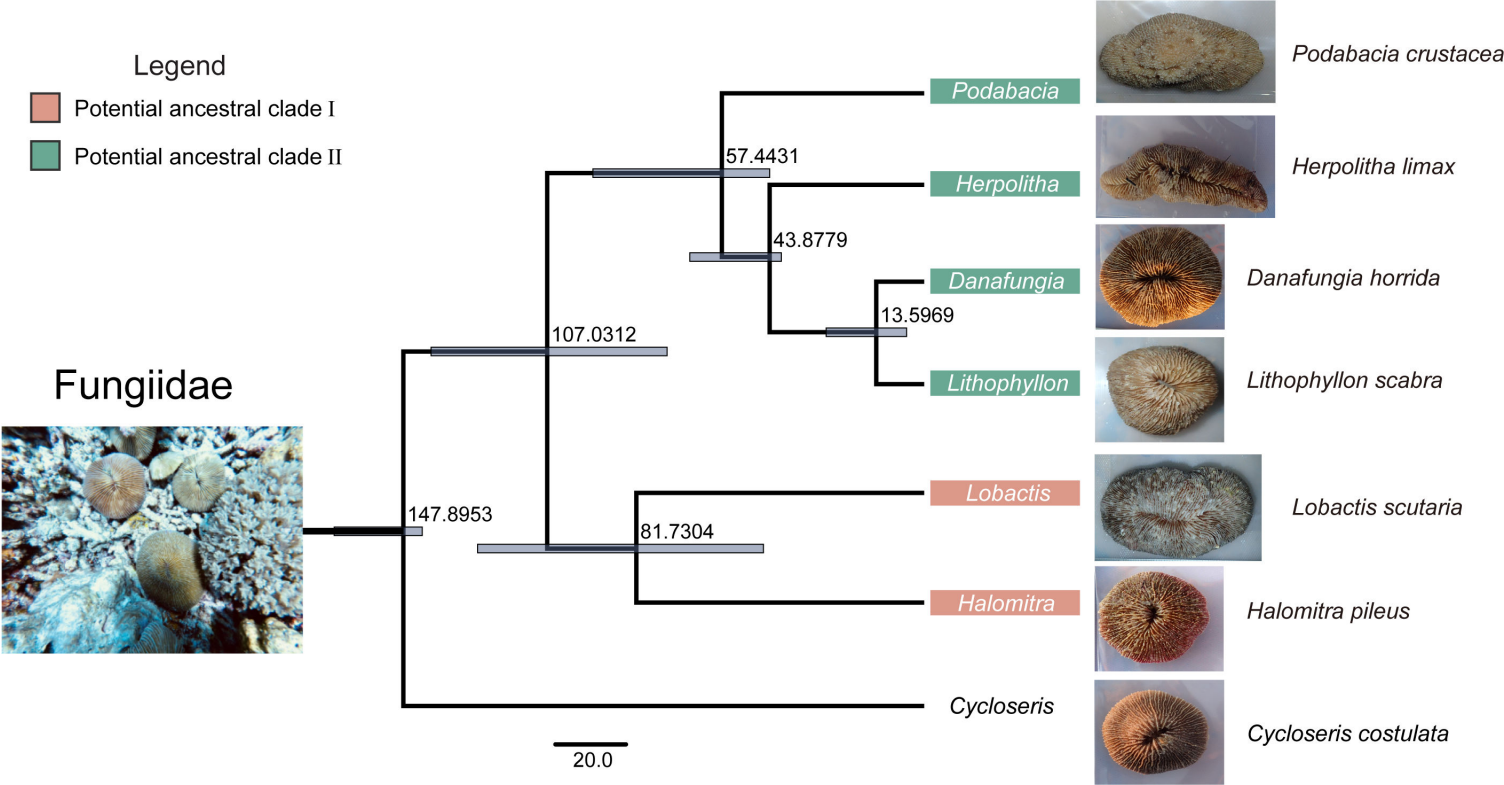
The divergence time estimation of Fungiidae at the genus level. The purple portions represent 95% of the highest probability density. Orange means potential ancestral clades I; green means potential ancestral clades II. Unit: million years (Ma).

The time calibration nodes used in this study were the earliest ages recorded by the fossils of *Cycloseris* and *Lithophyllon*, which were 136.4 and 37.2 Ma, respectively. The molecular clocks of Fungiidae were examined using Tracer, and the effective sample size value of all parameters was >200. Molecular clock analysis showed (Fig. 2) that *Cycloseris* was the older evolutionary branch of Fungiidae, which differentiated before 147.8953 Ma and was earlier than the other six genera. Fungiidae were differentiated into two ancestral clades before 107.0312 Ma. Among them, the divergence times of *Lobactis* and *Halomitra* in the potential ancestral clade I were estimated to have occurred 81.7304 Mya. In addition, *Podabacia* was the earliest divergent genus in the potential ancestral clade II, with a divergence time estimated at 57.4431 Ma, whereas the divergence time of *Herpolitha* was 43.8779 Ma. The latest genera to evolutionarily diverge were *Lithophyllon* and *Danafungia*, whose divergence times were estimated to be before 13.5969 Ma.

### Symbiodiniaceae diversity and bacteria diversity

**FIG 3:**
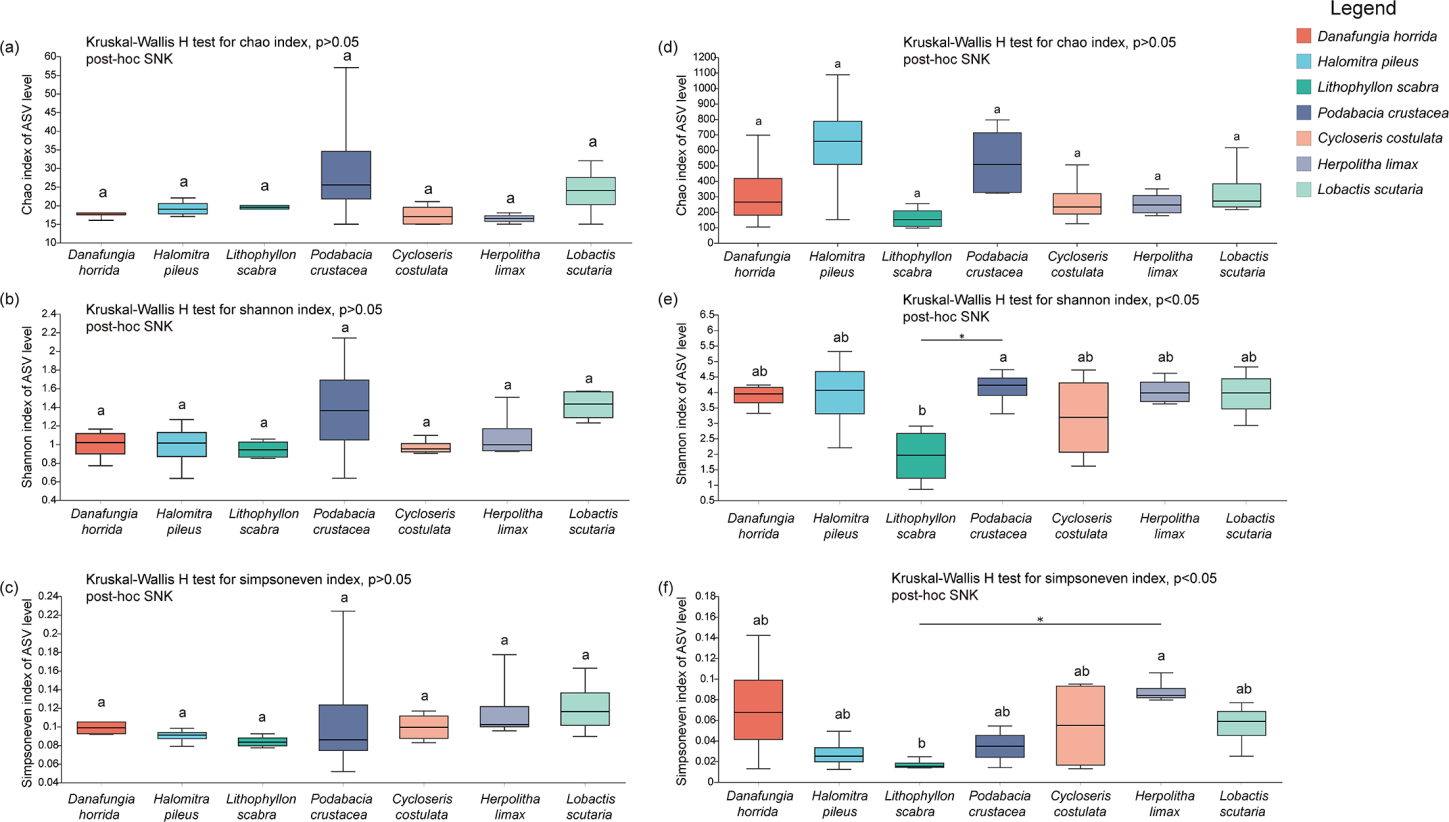
The α-diversity of Symbiodiniaceae and bacteria of Fungiidae at ASV level in SJR in the SCS. Symbiodiniaceae: (**a**) Chao index; (**b**) Shannon diversity index; (**c**) Simpson evenness index. Bacteria: (**d**) Chao index; (**e**) Shannon diversity index; (**f**) Simpson evenness index. Kruskal-Wallis *H* test.

Generational sequencing based on the ITS2 gene showed that 530,740 reads met quality control requirements. Based on the principle of standardized data analysis, quality-controlled reads were clustered into 154 valid amplicon sequence variants (ASVs) after flattening the samples according to the minimum number of sample sequences (*n* = 15,164). α-Diversity analysis showed that the Chao, Shannon diversity, and Simpson evenness indices did not differ significantly among the different coral species (Kruskal-Wallis *H* test, *P* > 0.05; Fig. 3a through c).

Moreover, after quality control of the 16S rRNA sequences, a total of 256,116 valid reads were identified. Based on the minimum number of sample sequences (*n* = 9,147), 5,797 valid bacteria ASVs clustered after flattening the sequence. There was no significant difference in the bacterial Chao index among the different coral species (Kruskal-Wallis *H* test, *P* > 0.05; Fig. 3d). However, the Shannon diversity index and Simpson evenness index of the bacteria varied significantly among the different coral species. The bacterial Shannon diversity index was significantly higher for *Podabacia crustacea* than for *Lithophyllon scabra* (Kruskal-Wallis *H* test, *P* < 0.05; Fig. 3e), whereas the Simpson evenness index of *Herpolitha limax* was significantly higher than that of *L. scabra* (Kruskal-Wallis *H* test, *P* < 0.05; Fig. 3f).

### Symbiodiniaceae community composition and community structure

**FIG 4:**
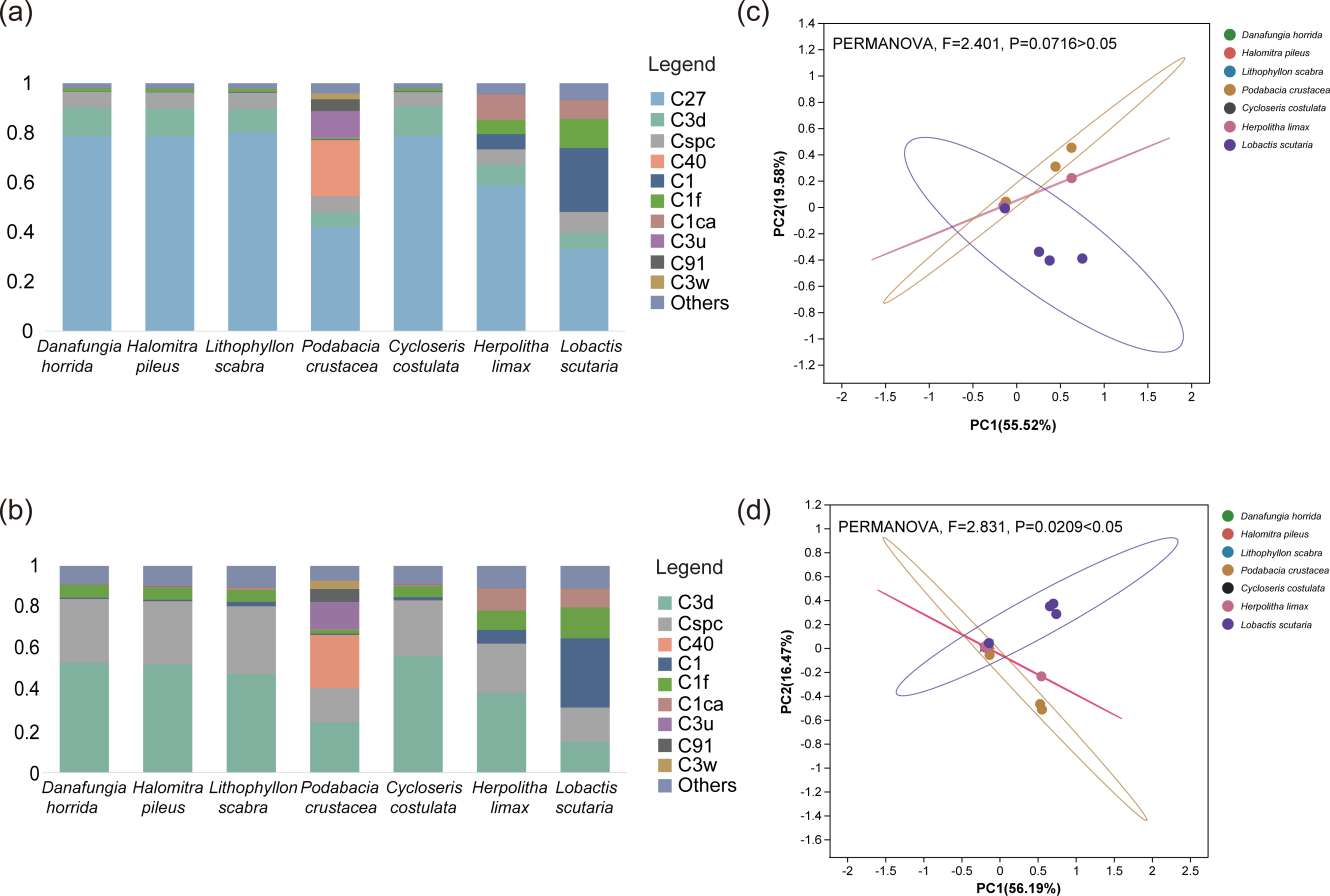
The community composition and community structure of Symbiodiniaceae of Fungiidae in SJR in the SCS. (**a**) The composition of the dominant/subdominant Symbiodiniaceae sub-clades (relative abundance >5% in at least one sample). (**b**) The composition of the subdominant Symbiodiniaceae sub-clades. Differences in Symbiodiniaceae community structure were examined *via* PCoA and PERMANOVA: (**c**) dominant/subdominant Symbiodiniaceae sub-clades; (**d**) subdominant Symbiodiniaceae sub-clades.

Ten dominant/subdominant Symbiodiniaceae sub-clades were identified in this study (relative abundance >5% in at least one sample; Fig. 4a), all of which belonged to *Cladocopium*. Notably, the Symbiodiniaceae communities of Fungiidae at SJR were all dominated by the C27 sub-clade, and the average relative abundance of C27 among the coral species was 64.4% ± 28.5% with high specificity. However, variations were observed in the composition of the subdominant Symbiodiniaceae sub-clades of Fungiidae (Fig. 4b). Among them, C3d and Cspc were dominant in the subdominant Symbiodiniaceae sub-clades of *Danafungia horrida* (C3d: 53.2% ± 5.9%; Cspc: 30.8% ± 3.3%), *Halomitra pileus* (C3d: 52.3% ± 4.6%; Cspc: 30.6% ± 3.0%), *Cycloseris costulata* (C3d: 56.4% ± 1.8%; Cspc: 26.8% ± 3.5%), *Lobactis scutaria* (C3d: 47.7% ± 3.5%; Cspc: 32.7% ± 4.1%), and *Herpolitha limax* (C3d: 38.6% ± 26.4%; Cspc: 23.7% ± 16.6%). However, the subdominant Symbiodiniaceae of *Lithophyllon scabra* contained a higher abundance of C1 (33.4% ± 20.8%), whereas the composition of *Podabacia crustacea* had a higher abundance of C40 (25.6% ± 38.0%) and C3u (13.2% ± 17.8%). In addition, principal coordinate analysis (PCoA) and permutational multivariate analysis of variance (PERMANOVA) analyses indicated that the community structure of the Symbiodiniaceae sub-clades did not vary markedly among the coral species (PERMANOVA, *F* = 2.401, *P* = 0.0716 > 0.05; Fig. 4c). However, in the absence of the dominant C27 sub-clade, the community structure of the subdominant Symbiodiniaceae sub-clades differed significantly among the coral species (PERMANOVA, *F* = 2.831, *P* = 0.0209 < 0.05; Fig. 4d).

### Bacteria community composition and community structure

**FIG 5:**
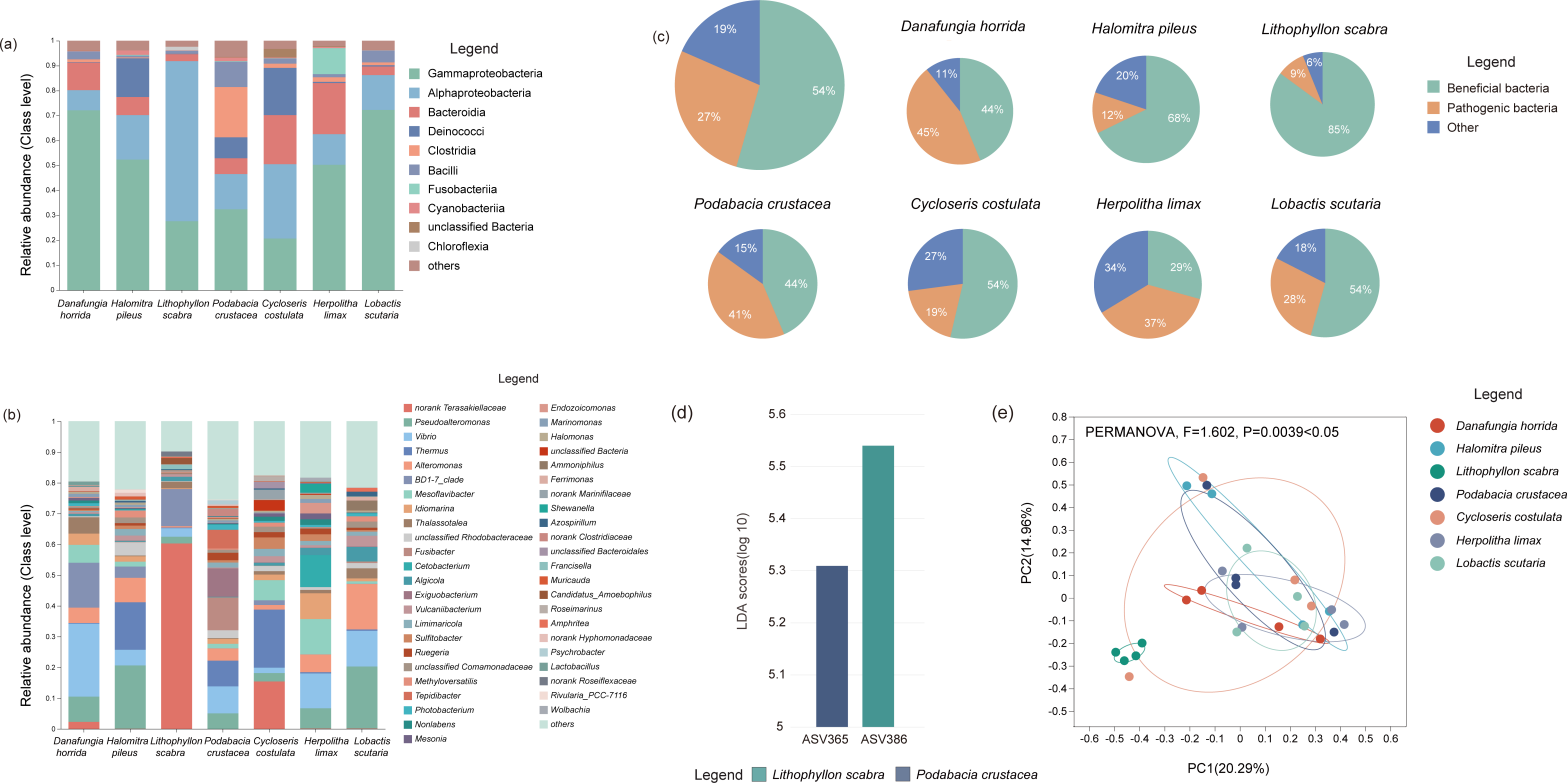
The community composition and community structure of bacteria of Fungiidae in SJR in the SCS. The histogram of bacteria community composition (others < 0.01): (**a**) at class level; (**b**) at genus level. (**c**) the relative abundance of representative beneficial bacteria, pathogenic bacteria, and unknown types of bacteria at the genus level (relative abundance >1%). (**d**) Enrichment characteristics of bacterial ASVs in Fungiidae. (**e**) Differences in bacterial community structure were examined *via* PCoA and PERMANOVA.

Regarding the bacterial community composition of Fungiidae, there were 10 bacteria with a relative abundance >1% at the class level (Fig. 5a). These included γ-proteobacteria (46.82% ± 20.76%), α-proteobacteria (22.90% ± 19.44%), Bacteroidia (10.15% ± 7.31%), Deinococci (6.34% ± 8.07%), Clostridia (3.77% ± 7.25%), Bacilli (3.30% ± 3.34%), Fusobacteriia (1.6% ± 3.88%), Cyanobacteria (0.65% ± 0.67%), unclassified bacteria (0.59% ± 1.25%), and Chloroflexia (0.21% ± 0.54%). Among these, γ-proteobacteria were dominant in *L. scutaria* (72.26%), *D. horrida* (72.11%), *H. pileus* (52.34%), *H. limax* (50.25%), and *P. crustacea* (32.41%). In contrast, α-proteobacteria were dominant in *L. scabra* (64.18%) and *C. costulata* (29.80%). It is important to note that the relative abundance of Deinococci was higher in *C. costulata* (18.95%) and *H. pileus* (15.54%) than in other coral species. Clostridia and fusobacteria were relatively abundant in *P. crustacea* (20.15%) and *H. limax* (10.40%), respectively.

At the genus level, 46 bacterial genera had an average relative abundance of >1% (Fig. 5b). Norank Terasakiellaceae (11.17% ± 22.36%), *Pseudoalteromonas* (9.36% ± 7.81%), *Vibrio* (9.28% ± 7.47%), *Thermus* (6.29% ± 8.05%), and *Alteromonas* (5.62% ± 4.77%) dominated in all samples. Notably, norank Terasakiellaceae (60.26%) were more abundant in *L. scabra*, and *C. costulata* had a greater abundance of *Thermus* (18.89%). Nevertheless, the abundances of *Fusibacter* (10.69%) and *Exiguobacterium* (9.23%) were higher in *P. crustacea*, and a higher abundance of *Mesoflavibacter* (11.48%) was found in *H. limax*. Moreover, *Vibrio* and the BD1-7 clade were the dominant bacteria in *D. horrida*, with relative abundances of 23.73% and 14.60%, respectively. Further analysis revealed that coral-beneficial bacteria represented by the norank Terasakiellaceae, *Pseudoalteromonas*, *Thermus*, *Alteromonas*, and BD1-7 clades accounted for 54% of the bacterial community in all coral samples. *Vibrio*, *Mesoflavibacter*, *Thalassotalea*, *Fusibacter*, and *Algicola*, which on behalf of the pathogenic bacteria accounted for 27%, and other unknown-type bacteria accounted for 19% (Fig. 5c; Table S2). The relative abundances of beneficial bacteria were 85% and 68% for *L. scabra* and *H. pileus*, respectively, whereas the abundance was 54% for both *C. costulata* and *L. scutaria*. Potentially pathogenic bacteria dominated the bacterial community of *H. limax* (37%), and their relative abundance was higher than that of beneficial bacteria (29%; Fig. 5c).

Additionally, the results of linear discriminant analysis (LDA) effect size (LEfSe) analysis based on bacterial ASVs (Fig. 5d) indicated that the two bacterial ASVs exhibited differentially enriched characteristics (LDA >2.0; *P* < 0.05) in the bacterial communities of *L. scabra* and *P. crustacea*, respectively, and both belonged to α-proteobacteria. Further analysis suggested that ASV365 belongs to *Ruegeria* (*Ruegeria lacuscaerulensis*) and ASV386 belongs to the norank Terasakiellaceae. Analyses of PCoA and PERMANOVA revealed (Fig. 5e) that the bacterial community structure of Fungiidae varied significantly among the coral species (PERMANOVA, *F* = 1.602, *P* = 0.0039 < 0.05). Compared to other coral species, the bacterial community structure was more discrete in *C. costulata*, while the bacterial community of *L. scabra* had a higher homogeneity.

### Core bacterial microbiome

**FIG 6:**
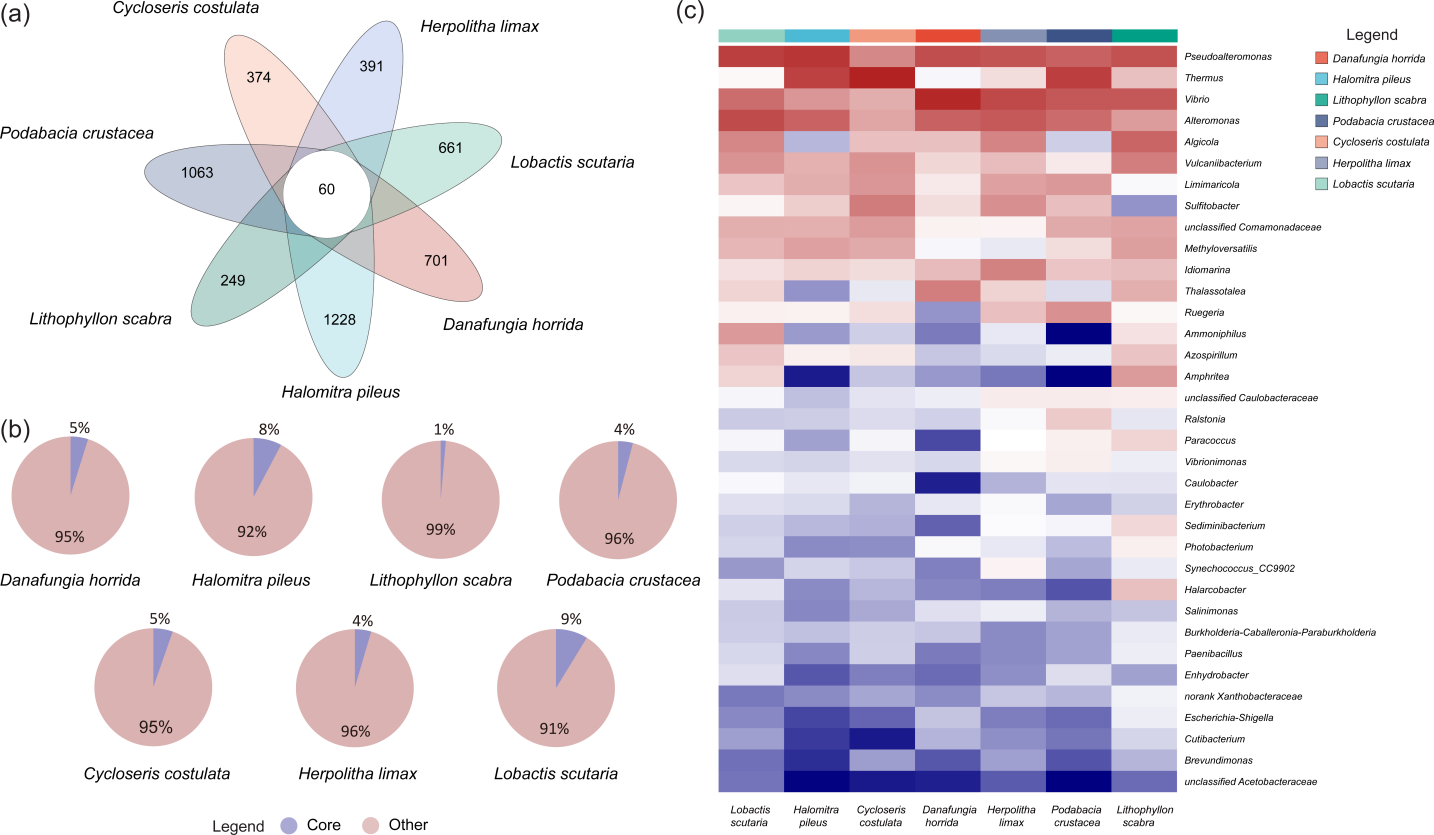
The community composition of the core bacterial microbiome of Fungiidae in SJR in the SCS. (**a**) Venn analysis at the ASV level. (**b**) The percentage of core microbiome in coral bacterial community composition. (**c**) The heat map of the core microbiome at the genus level.

According to the Venn analysis, 60 bacterial ASVs were found in all Fungiidae samples (Fig. 6a). These ASVs constitute the core bacterial microbiome of Fungiidae in the SCS, involving 7 phyla, 8 classes, 19 orders, and 35 genera (Table S3). The relative abundances of the core bacterial microbiome of each coral species varied from 1% to 9%, which belonged to the dominant bacterial type (Fig. 6b). At the class level, Gammaproteobacteria (*n* = 40), Alphaproteobacteria (*n* = 12), Bacilli (*n* = 2), Bacteroidia (*n* = 2), Actinobacteria (*n* = 1), Campylobacteria (*n* = 1), Cyanobacteria (*n* = 1), and Deinococci (*n* = 1) were identified. At the genus level, the core bacterial microbiome of *H. pileus* had a higher abundance of *Thermus* (33.30%) and *Pseudoalteromonas* (23.61%), whereas *Pseudoalteromonas* (24.81%) and *Alteromonas* (19.80%) were higher in *L. scutaria*. Furthermore, *Thermus* (21.67%), *Sulfitobacter* (13.42%), and *Pseudoalteromonas* (13.28%) were the dominant types of core bacterial microbiomes in *C. costulata*, while *L. scabra* was dominated by *Vibrio* (19.16%), *Pseudoalteromonas* (15.54%), and *Vulcaniibacterium* (13.09%). Notably, *Vibrio* was stably present in the core bacterial microbiome of Fungiidae and dominated *D. horrida*, *L. scabra*, *P. crustacea*, and *H. limax*, with relative abundances of 34.13%, 19.16%, 24.84%, and 22.95%, respectively.

### The microbial interaction network of Symbiodiniaceae and bacteria

**FIG 7:**
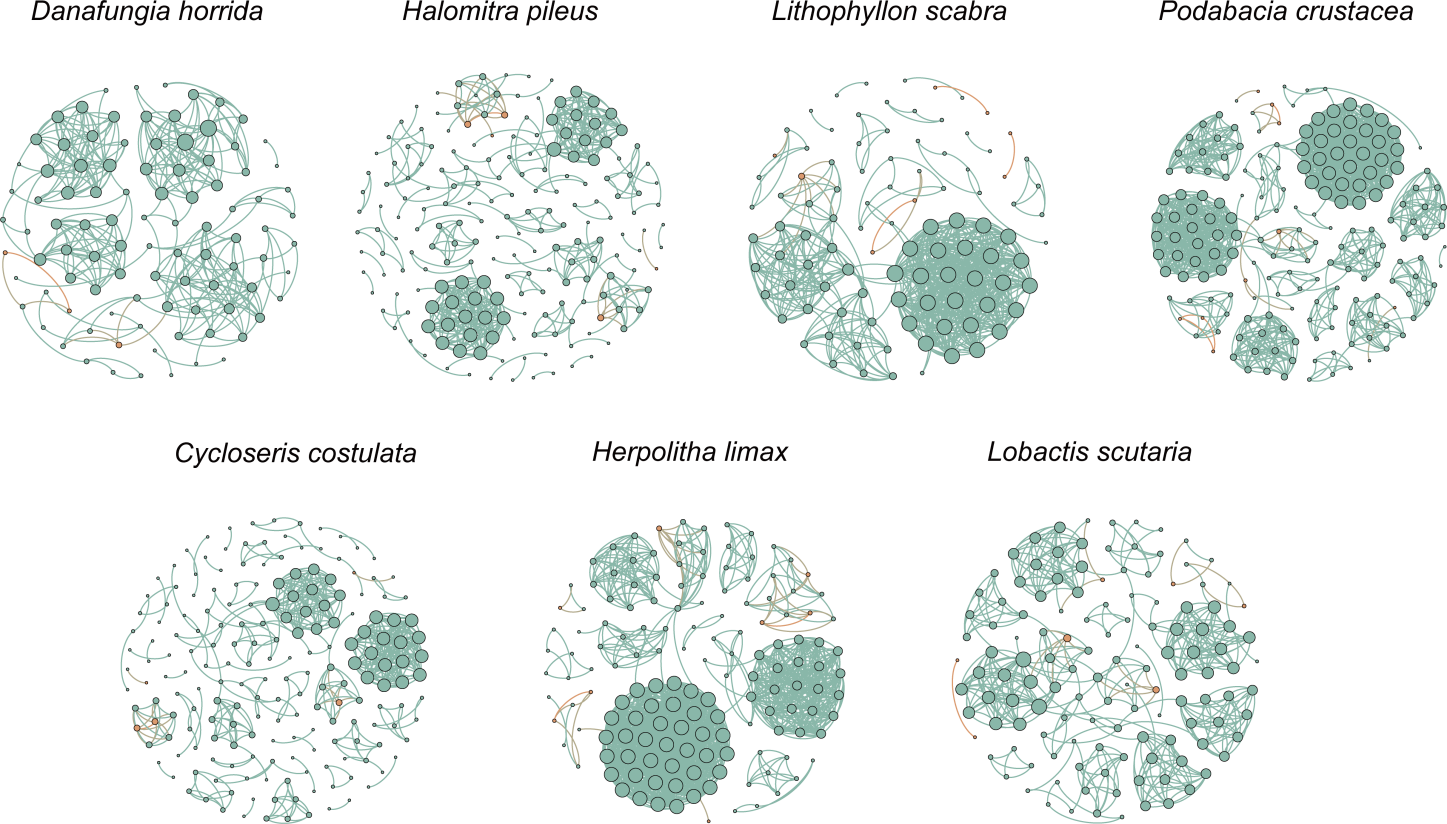
The microbial interaction network of Symbiodiniaceae and bacteria of Fungiidae in SJR in the SCS. Only co-occurrence correlations were shown in the figure. The dominant Symbiodiniaceae sub-clades were denoted by orange, and bacteria were denoted by green.

There were diverse interactions between the dominant Symbiodiniaceae sub-clades (relative abundance >5%) and the bacteria (Fig. 7). The nodes of the microbial interaction network varied between 105-188, and the number of interaction correlations varied between 443 and 1617. The number of co-occurrence correlations changed between 306 and 1,488. *C. costulata* had the highest number of co-occurrence correlations, accounting for 92% of the total interactions. *H. limax*, *P. crustacea*, and *L. scabra* exhibited higher co-occurrence correlations. The quantities were 1,210, 1,214, and 556, accounting for 90%, 85%, and 80% of their total number of interactions, respectively. In contrast, *D. horrida*, *L. scutaria*, and *H. pileus* exhibited 306, 639, and 640 co-occurrence correlations, respectively. These correlations constituted 69%, 68%, and 66% of their total interactions, respectively. Thus, co-occurrence correlations were the main mode of interaction for Fungiidae.

In addition, the topological properties of Fungiidae differed among potential evolutionary branches (Table 1). Potential ancestral clade I had higher complexity (I = 7.046; II = 6.829), network diameter (I = 8; II = 7.25), average degree (I = 23.029; II = 22.330), and average path length (I = 3.160; II = 2.411). Potential ancestral clade II had a higher clustering coefficient (I = 0.591; II = 0.812), network centralization (I = 0.171; II = 0.237), modularity (I = 0.43; II = 0.476), and betweenness centrality (I = 0.19; II = 0.0245). These results suggest that the microbial interaction network of coral species in the potential ancestral clade II, which later underwent evolutionary divergence, had lower complexity and higher resilience than the potential ancestral clade I of Fungiidae.

**TABLE 1:** The attributions and topological properties of the Symbiodiniaceae-bacteria interaction network

Species		Nodes	Edges	Number of co-occurrence correlations	Complexity	Network diameter	Network centralization	Average path length	Average degree	Clustering coefficient	Modularity	Betweenness centrality
*Cycloseris costulata*		141	1,617	1,488	11.468	6	0.364	1.800	25.352	0.890	0.558	0.018
*Lobactis scutaria*	Potential ancestral clades I	113	940	639	8.319	11	0.139	4.043	18.872	0.748	0.554	0.023
*Halomitra pileus*		168	970	640	5.774	5	0.203	2.276	27.185	0.433	0.306	0.015
*Podabacia crustacea*	Potential ancestral clades II	188	1,425	1,214	7.580	13	0.194	3.803	33.841	0.802	0.392	0.023
*Herpolitha limax*		149	1,346	1,210	9.034	5	0.294	1.703	27.101	0.877	0.421	0.013
*Danafungia horrida*		109	443	306	4.064	6	0.154	2.116	13.633	0.721	0.565	0.035
*Lithophyllon scabra*		105	697	556	6.638	5	0.306	2.02	14.743	0.847	0.526	0.027

## DISCUSSION

### Thermal tolerance of Fungiidae is regulated by evolutionary radiation, specificity symbiosis, and subdominant Symbiodiniaceae taxa

The results of the analyses showed that the α-diversity and community structure of Symbiodiniaceae in SJR did not differ significantly among the coral species. However, the results of community composition analyses showed that Fungiidae had a highly specific symbiotic relationship with the C27 sub-clade. This indicates that the C27 sub-clade may serve as the optimal symbiotic partner, establishing a stable symbiotic relationship with *C. costulata* before evolutionary divergence began. Previous phylogenetic and molecular clock analyses of Symbiodiniaceae were conducted based on LSU rDNA and mitochondrial cytochrome b genes. These analyses indicated that the origin and evolutionary divergence of Symbiodiniaceae took place during the Middle-Late Jurassic period, approximately 140–200 Mya (28). This timeframe aligns with the evolutionary divergence time of the relatively ancient C. costulata (about 147 Mya). The recognition of Symbiodiniaceae by juvenile corals is mostly non-selective, allowing hosts to establish symbiotic relationships with multiple types of Symbiodiniaceae (72). However, there are a series of winnowing processes during the development of the coral host, which would help the coral host select the most suitable symbiotic partner among diverse symbiotic types (73–75). In addition, Fungiidae have a unique survival strategy, with most species being able to migrate between habitats during the free-living phase (59, 76, 77). This unique mobility may improve genetic connectivity between coral hosts and provide more opportunities for sharing symbionts (78). In addition, the C27 sub-clade may potentially enhance the heat tolerance of Fungiidae (79). Former studies have shown that *Podabacia* and *Pleuractis* were all specifically symbiotic with the C27 sub-clade in the tropical reefs of southern Singapore and northeastern Peninsular Malaysia (80, 81). Similarly, following the global coral thermal bleaching event in 2015–2016, Fungiidae in Panshi (Xisha Islands) exhibited a more stable composition of Symbiodiniaceae, with Symbiodiniaceae in both the outer reef slope and the lagoon dominated by the C27 sub-clade (65). This evidence suggests that specific symbiosis with the C27 sub-clade may effectively enhance the adaptation of Fungiidae to high-temperature environments.

Community composition analysis revealed interspecific variation in the composition of the subdominant Symbiodiniaceae sub-clades, which was closely related to the evolutionary radiation of Fungiidae. Different types of Symbiodiniaceae confer diverse heat-adaptive potential on coral holobionts (29–31). In the potential ancestral clade I, the subdominant Symbiodiniaceae of *H. pileus* was dominated by Cspc and C3d, but *L. scutaria* contained a higher abundance of C1. The C1 sub-clade is commonly distributed at relatively high latitudes in the SCS, with strong photosynthetic efficiency and sensitivity to thermal stress (34, 82). The results of an experiment on *Acropora tenuis* subjected to heat stress (25–30°C) showed that corals with clade D survived 100% throughout the test period. In comparison, the survival of corals with C1 dropped to 77% by only the eighth day of the experiment (83). Similarly, the *A. millepora* with C1 was sensitive to elevated water temperatures. When the temperature rose to 34°C, the Fv/Fm values of C1 decreased sharply, leading to coral death after 4 days. In contrast, the Fv/Fm value of clade D, which was symbiotic with *Galaxea fascicularis*, decreased but still maintained around 0.6, indicating a high thermal tolerance (84). Regarding the potential ancestral clade II, the subdominant Symbiodiniaceae of *H. limax*, *D. horrida*, and *L. scabra* were also dominated by Cspc and C3d, but *P. crustacea* contained a higher abundance of C40 and C3u. C3u is a heat-tolerant sub-clade of Symbiodiniaceae, and the symbiotic relationship between C3u and corals generally results in strong thermal adaptability (34, 66, 85). A high abundance of C3u has been found in tropical coral reef communities, such as the western Indian Ocean (66), Andaman Sea, Gulf of Thailand (66), Philippines Archipelago (86, 87), and South China Sea (34, 79). Along the direction from relatively high-latitude to low-latitude coral reefs in the SCS, Chen et al. observed a shift in the community composition from C1 (relative abundance 29%) to C3u (relative abundance 47%) in the dominant Symbiodiniaceae of *Acropora formosa* (34). In the same way, the community composition of the dominant Symbiodiniaceae of *Favia palauensis* also showed the change of latitude gradient, which transformed the C1 on Weizhou Island (21°00′–21°04′N, 109°04′–109°08′E) into the C3u on Xinyi Reef (9°20′–9°21′N, 115°54′–115°58′E) (34, 79). This implies that C3u may improve the resistance of corals to high-temperature environments at low latitudes in the SCS. Moreover, Sikorskaya et al. used chromatography with high-resolution mass spectrometry to identify the lipidome profiles of the thylakoid membranes of Symbiodiniaceae. They found that C3u may be responsible for the expression of thermotolerant lipidome features in the coral holobiont, which are important for the stability and resilience of corals in response to thermal stress (85). In the case of C40, a phylogeny study revealed that it is similarly related to C3u, both stemming from the C3 branch (27, 34). Symbiodiniaceae sub-clades with more intimate phylogenetic relationships in *Cladocopium* may have similar environmental adaptations (34). For example, analyses of phylogeny and canonical correspondence analysis based on coral holobionts in the SCS showed that the relative abundance of Symbiodiniaceae sub-clades with C3 as a common ancestor (C3u, Cspc, C40, C27, etc.) was positively correlated with SST, photosynthetically available radiation, and salinity. In contrast, the opposite was true for sub-clades with C1 as a common ancestor (C1ca, C1p, C1c, etc.) (34). This suggests that C40 may possess heat tolerance similar to that of C3u and may play a potential role in enhancing the thermal adaptability of corals.

In summary, C27 sub-clades may have established a stable and specific symbiotic relationship with Fungiidae before they began to diverge evolutionarily and could potentially play a role in enhancing the heat tolerance of Fungiidae. In addition, the community composition of the subdominant Symbiodiniaceae sub-clades was strongly associated with the thermal tolerance of Fungiidae in different ancestral clades. The community composition of *L. scutaria* (potential ancestral clade I; 81.7304 Ma) contained a higher abundance of C1, which may have enhanced its sensitivity to thermal stress. In contrast, *P. crustacea* (potential ancestral clade II: 57.4431 Ma) had a higher abundance of potentially thermotolerant C40 and C3u. Therefore, this study suggests that the thermal tolerance of Fungiidae is regulated by evolutionary radiation, specific symbiosis, and sub-dominant Symbiodiniaceae taxa.

### The evolution of Fungiidae enhances the stability of bacterial communities and the thermal adaptation of coral holobionts

The results of molecular clocks suggest that the evolutionary divergence between *C. costulata* (*Cycloseris*) and six other genera occurred approximately 147.8953 Mya. In contrast, the divergence time of *L. scabra* and *D. horrida* was relatively recent, approximately 13.5969 Mya. It is worth noting that the settings of various models and parameters (e.g., substitution models, molecular clock models, and fossil calibration points) in the process of molecular clock analysis may introduce errors, which may affect the reliability of the evolutionary divergence time estimation. Although the choice of parameters was duly considered in this study, the calculated results may still differ from the true divergence time of Fungiidae. In addition, the results of the bacterial Shannon diversity index and PCoA analysis showed that, in SJR, *L. scabra* had lower α-diversity and a more stable community structure than other coral species. In comparison, *C. costulata* had higher β-diversity and a relative dispersion community structure. These results suggest that the regulation ability of the microbial community of corals was increased during the evolutionary divergence of Fungiidae, which may have been associated with the gradual maturation of functions related to the immune system and enhanced the stability of the bacterial community and thermal adaptation of the coral holobiont. Scleractinian corals have undergone tremendous environmental changes during their evolution, with the primary threat being abnormal SST caused by climate change (7, 8). The microbial interaction and symbioses of coral holobiont develop novel strategies and diversify as coral species evolve, which is key to the ability of scleractinian corals to survive successfully in the context of increased climate change (47, 88). The dynamics of coral bacterial communities are strongly associated with coral development (18). Juvenile corals typically have more diverse bacterial microbiomes than adult corals (89, 90). Littman et al. found that the Shannon-Weaver diversity index (4.8), Chao’s richness estimator (474.2), and Simpson’s evenness (238.2) of juvenile *A. millepora* are higher than those of adult corals (three indices: 3.4, 80.2, and 21.74, respectively) (90). This may be explained by the underdevelopment of functions associated with the juvenile corals’ immune system (89). In addition, stressed coral colonies have higher α-diversity and β-diversity than healthy coral colonies (45, 91–93). Diazotroph diversity (Chao1, Shannon) in *Mussismilia harttii* significantly increased threefold after experiencing high-temperature treatments (+2.5°C and +4°C) (40). In addition, the bacterial diversity of *Pocillopora damicornis* subjected to heat stress (31°C) was significantly higher (Chao1: 1,406 ± 155 SD) than that of the control group (Chao1: 995 ± 23 SD; *P* < 0.05) (94). However, increased diversity does not indicate enhanced thermal adaptation in corals; rather, it likely indicates that heat stress reduces the ability of the coral host to regulate the bacterial community (45). McDevitt-Irwin et al. revealed that high temperatures decrease the abundance of beneficial bacteria (e.g., *Endozoicomonas*) and increase the abundance of pathogenic bacteria (e.g., Flavobacteriales, Rhodobacterales, and Vibrionales), causing an increase in the α-diversity of coral bacterial communities (45). Coral bacterial communities also exhibit high β-diversity under thermal stress conditions (93). For instance, elevated temperatures cause opportunistic bacteria (e.g., Proteobacteria) to proliferate and destabilize coral bacterial communities, increasing β-diversity (95). This suggests that inhibition of pathways associated with coral immunity by heat stress leads to increased variation among coral bacterial communities (93, 96). Thus, *L. scabra*, with lower bacterial α-diversity and a stable community structure, indicated a possible strong thermal tolerance, whereas *C. costulata* with higher β-bacterial diversity and an unstable community structure showed a weaker regulation capacity of the microbial community and higher sensitivity to the thermal environment. Moreover, corals can increase their adaptability and resilience to thermal stress by removing dysfunctional or favorable symbiotic relationships through the immune system to maintain balance and stability in the relationship between coral hosts and their microbiome (46, 97). Hence, *L. scabra* with later differentiation time had stable bacterial communities and heat-tolerant coral holobionts, probably because of the continuous evolution of the immune system driven by heat stress.

In conclusion, this study speculates that there is a close relationship between bacterial diversity, community structure, and the evolution of Fungiidae. Corals that differentiate later may improve regulation ability in bacterial communities, thereby enhancing the thermal adaptability of coral holobionts.

### The heat tolerance of Fungiidae is shaped by beneficial bacteria and the core bacterial microbiome

The results of the bacterial community composition showed that the beneficial bacteria represented by the norank Terasakiellaceae, *Pseudoalteromonas*, *Thermus*, *Alteromonas*, and BD1-7 clade accounted for 54% of the bacterial community in SJR in the SCS. This may be one of the main reasons why Fungiidae became the “winner” in the third global coral bleaching event. Beneficial bacteria are closely associated with the health of coral holobionts and play an important role in improving their thermal tolerance (38, 98–100). For example, beneficial bacteria (including *Pseudoalteromonas*, *Cobetia*, and *Halomona*s) help *P. damicornis* to alleviate the stress caused by high temperatures and reduce the degree of coral bleaching (101). Some strains of the BD1-7 clade may use proteorhodopsin to harvest light as an additional source of energy for corals (102, 103) to enhance the stability and resilience of corals in response to heat stress. With regard to the typical heat-tolerant *Thermus*, Qin et al. found that in tropical coral reef areas of the SCS (Panshi, Xisha Islands), some coral bacterial communities (e.g., *Pocillopora verrucosa*, *Pachyseris rugosa*, *Porites lutea*, *F. palauensis*, and *Favites abdita*) have a higher abundance of *Thermus* in the thermal lagoon environment. This abundance was generally higher than that observed on the outer reef slope (65), suggesting that *Thermus* may contribute to enhancing the ability of corals to adapt to hot environments. Furthermore, the beneficial bacteria of the family Fungiidae play an important role in the nitrogen and sulfur cycling processes of coral holobionts. Certain members of Terasakiellaceae have been proven to be nitrogen fixers (104) and are involved in nitrogen cycling processes (105), especially in habitats with significant food restrictions, such as the deep sea (104). In addition, many members of *Alteromonas* can provide nitrogen sources for the Symbiodiniaceae of coral larvae (106) and participate in the sulfur-cycling process of coral holobionts (107). This suggests that a bacterial community composition dominated by beneficial bacteria may help improve the ability of Fungiidae to adapt to high-temperature environments in the southern SCS.

In addition, the results of the LEfSe analysis showed that the beneficial bacteria *Ruegeria lacuscaerulensis* (*Ruegeria*) and norank Terasakiellaceae exhibited enrichment characteristics in the bacterial communities of *P. crustacea* and *L. scabra*, respectively, which indicated that the coral holobionts of *P. crustacea* and *L. scabra* may depend on beneficial bacteria to adapt to the high-temperature stress in SJR in the SCS. In hot environments, the relative abundance of *Vibrio* in corals increases rapidly, leading to a reduction in thermal acclimation (94). *Ruegeria* is a potential probiotic for corals, playing a role in protecting them from *Vibrio* (108) and in mutualism with Symbiodiniaceae (109). By isolating and culturing bacteria associated with *Galaxea fascicularis*, Miura et al. found three *Ruegeria* sp. strains capable of inhibiting the activity of *Vibrio coralliilyticus* (108). Ruegeria TM1040 forms a biofilm on the surface of *Pfiesteria* sp., improving its nutrient uptake (109). Thus, for *P. crustacea*, the increase in the abundance of beneficial *Ruegeria* may inhibit the activity of *Vibrio* and assist in the construction of biofilms by Symbiodiniaceae, thereby reducing the susceptibility of corals to thermal bleaching. Although one study noted that *Ruegeria* is closely associated with *Ctenactis crassa* and *Herpolitha limmax* infections and yellow-band disease (1,10). Terasakiellaceae also positively contributed to the thermal adaptation of the corals. Parker et al. found that Terasakiellaceae is stably present in *Orbicella faveolata* in Mermaid Reef in Great Abaco, which has a strong thermal tolerance (heat-tolerant threshold up to 33.0°C) (111). This implies that Terasakiellaceae, which were enriched in the bacterial community, may contribute to improving the thermal adaptability of *L. scabra*.

The core bacterial microbiome is closely related to the thermal adaptability of coral holobionts (112). Based on ASV analysis, 60 bacterial ASVs were identified as the core bacterial microbiome of Fungiidae in SJR. *Vibrio*, a typical coral pathogen (43, 113), exists stably in the core bacterial microbiome of Fungiidae. This suggests that there may be a potential threat to the health of Fungiidae in SJR in the SCS. Previous studies have shown that thermal stress can destabilize the symbiotic relationship between corals and bacteria, leading to a large decrease in the species and relative abundance of beneficial bacteria in coral bacterial communities, whereas the relative abundance of *Vibrio* rises rapidly (43, 94). For instance, heat stress experiments have shown that the abundance of *V. coralliilyticus* in *P. damicornis* increased by four orders of magnitude with increasing temperature (94). Bourne et al. also discovered that the proportion of *Vibrio*-associated clones in the bacterial community of *A. millepora* increase during thermal bleaching events (43). In addition, studies on the bacterial communities of *Cladocora caespitosa* and *Oculina patagonica* in the Mediterranean have shown that after experiencing anomalous summer temperature increases, the number of *Vibrio* in unhealthy corals is more than five times higher than that in healthy corals (114). However, a significant increase in *Vibrio* under abnormally high-temperature stress may increase the risk of coral infections with diseases (114–116). Many coral diseases related to *Vibrio* have been previously reported. *V. coralliilyticu*s was a causative agent of bleaching and tissue lysis in the tropical coral *P. damicornis* (117, 118) and has also been proven to be associated with coral bleaching and white syndromes in the Indo-Pacific (119,,^120^ ^120^,^120^). In addition, a consortium consisting of *Vibrio rotiferianus*, *Vibrio harveyi*, *Vibrio alginolytic*us, and *Vibrio proteolyticus* induced a yellow band disease infection in *Montastraea* spp. in Caribbean and *Diploastrea heliopora*, *Fungia* sp., and *Herpolitha* sp. in the Indo-Pacific (121, 122). This consortium directly attacked *Symbiodinium* spp. in coral tissues, resulting in a significant decrease in the mitotic index of Symbiodiniaceae, eventually leading to coral death (117). Accordingly, the stable presence of *Vibrio* in the core bacterial microbiome of Fungiidae may serve as an indicator of heat stress and coral host health, increasing the risk of thermal bleaching in Fungiidae in the southern SCS.

Therefore, this study infers that the potentially beneficial bacteria contribute to the heat tolerance of Fungiidae and are a crucial factor for Fungiidae to be winners during heat stress events; however, the stable presence of pathogenic bacteria in the core bacterial microbiome implies that Fungiidae may be potentially at risk for thermal bleaching, both of which together shape the heat tolerance of Fungiidae.

### The relationship between species evolutionary divergence and microbial interactions and its effect on the heat adaptive potential of Fungiidae

Molecular ecological network analysis revealed complex interactions between the dominant Symbiodiniaceae sub-clades and bacteria. Although the interaction direction between Symbiodiniaceae and bacteria in the molecular ecological network was difficult to determine, the proportion of co-occurrence correlations in the total number of interactions among the coral species varied from 66% to 92%, which implies that this relationship is the main interaction mode of the microbiome. This indicates the existence of a close cooperative relationship between Symbiodiniaceae and bacteria, which may be associated with the thermal adaptation potential of Fungiidae. There are complex interactions among microorganisms, including symbiosis, reciprocity, synergism, competition, predation, and parasitism (123–125). More frequent positive relationships in molecular ecological networks indicate a higher degree of cooperation between microbiomes (126), which can help coral holobionts adapt better to the pressure caused by high-temperature environments (127). It has been reported that *Endozoicomonas* had a close physical relationship with Symbiodiniaceae and directly protected Symbiodiniaceae from bleaching pathogens during thermal bleaching events (78, 128). Additionally, macromolecular compounds with nitrogen produced by Symbiodiniaceae must undergo bacterial metabolism and decomposition to supply coral hosts (129). Symbiodiniaceae is an important producer of dimethyl sulfate compounds in the coral antioxidant system (130) and may participate in the construction of bacterial communities with carbon and sulfur cycling in coral holobionts (107), which can maintain the stability of the material cycle of coral holobionts under heat stress and enhance the adaptability of corals to cope with thermal stress. From this, it can be seen that microbial interactions dominated by cooperation play an important role in maintaining the homeostasis and material metabolism of coral holobionts, which may be the driving force for the thermal tolerance of Fungiidae.

Analysis of topological properties revealed that the potential ancestral clade II had a lower average path length and network complexity and a higher clustering coefficient and betweenness centrality than the potential ancestral clade I of Fungiidae. This implies that the evolutionary divergence of Fungiidae may have influenced the interactions between Symbiodiniaceae and bacteria. Decreased complexity and increased betweenness centrality of microbial interaction networks may improve the heat tolerance of coral holobionts (127, 131, 132). Coral holobionts exhibit high microbial network complexity under high thermal stress (127, 131). For example, the results of the analysis of the bacterial interaction networks of 53 coral species (total of 26 genera) from Tiezhi Reef (11°03′N, 114°23′E) in Nansha Islands showed that the bacterial interaction networks of bleached corals (network nodes: 420; number of interactions: 745) were more complex than those of healthy corals (network nodes: 251; number of interactions: 468) (131). Similarly, corals subjected to heat stress (32°C) exhibited higher interaction network complexity than healthy *Porites cylindrica* (127). Furthermore, Chen et al. found a significant positive correlation between bacterial diversity and the complexity of microbial interaction networks (133). This correlation arises because high-temperature stress reduces the ability of the coral to regulate its microbiome. Additionally, it promotes the invasion of pathogenic and opportunistic bacteria, thus disrupting the homeostasis of coral holobionts (133, 134). Therefore, heat stress may increase the diversity of bacteria in corals, leading to an increase in the complexity of microbial interaction networks. Moreover, an increase in betweenness centrality implies that microbial interaction networks may have greater resilience because the removal of nodes does not greatly impact the connectivity of other nodes (132, 135). Lima et al. found that betweenness centrality for the microbial interaction networks of the surface mucus layer of *Pseudodiploria strigosa* is higher in the inner reef (annual temperature range of 13–15°C) than in the outer reef (annual temperature fluctuations of 10°C) in Bermuda (132). Hence, Symbiodiniaceae-bacterial interaction networks with high betweenness centrality may have greater resilience, enhancing the thermal adaptation potential of coral holobionts. Notably, the higher clustering coefficient and the lower average path length also suggested that coral species of potential ancestral clade II may have greater thermal adaptability (136, 137). The clustering coefficient was related to the stability and robustness of the microbial interaction network (138, 139). Under heat stress, the relative abundance of potentially opportunistic bacteria increases dramatically and dominates the bacterial community, causing corals to shift to an unstable state and induce bleaching (45, 140). The clustering coefficient of the microbial interaction network of *P. damicornis* was significantly reduced by 27.98% to 29.17% (*P* < 0.05) after the addition of AHLs and acyl-homoserine lactones, which can induce coral bleaching (140). The average path length measures the shortest distance between all nodes within a network. A shorter average path length indicated that the network nodes were more closely related and the transmission efficiency of information or energy in the network was higher (53, 137, 141). Consequently, the higher clustering coefficient and lower average path length indicated that the potential ancestral clade II may enhance its ability to adjust to heat stress by improving the stability and information/energy transfer efficiency of the microbial interaction network.

This evidence suggests that co-occurrence correlations are the primary mode of interaction for the microbiome of Fungiidae, suggesting that Fungiidae may cooperate through close microbial relationships to enhance the heat tolerance of coral holobionts. Furthermore, the evolutionary divergence of Fungiidae was strongly linked to microbial interactions. The microbial interaction network of coral species of potential ancestral clade II, which diverged later, possessed lower complexity, greater resilience, higher stability, and higher information/energy transfer efficiency, suggesting that they may possess greater heat tolerance. Nevertheless, the microbiome interaction mechanism or pattern between Symbiodiniaceae and bacteria in Fungiidae needs to be further verified using fluorescence *in situ* hybridization, omics, and co-culture experiments.

### Conclusions

This study found that *Cycloseris* was an older evolutionary branch of the Fungiidae (approximately 147.8953 Mya). Before 98.4252 Ma, Fungiidae differentiated into two potential ancestral clades. Potential ancestral clade I included *Lobactis* (81.7304 Ma) and *Halomitra* (81.7304 Ma), whereas potential ancestral clade II included *Podabacia* (57.4431 Ma), *Herpolitha* (43.8779 Ma), *Lithophyllon* (13.5969 Ma), and *Danafungia* (13.5969 Ma). Symbiodiniaceae diversity showed no significant differences in the Fungiidae. The community composition and structure of Symbiodiniaceae revealed that Fungiidae had a highly specific symbiotic relationship with the potentially thermotolerant C27 sub-clade; however, the community composition of the subdominant sub-clades of Symbiodiniaceae differed among species. The community composition of the Symbiodiniaceae of *L. scutaria* (*Lobactis*), belonging to the potential ancestral clade I, contained a higher abundance of the thermosensitive C1 sub-clade, while *P. crustacea* (*Podabacia*), belonging to the potential ancestral clade II, had a higher abundance of the thermotolerant C40 and C3u sub-clades. This implies that the thermal adaptation potential of Fungiidae is closely related to the specific symbiosis of potentially heat-tolerant Symbiodiniaceae, and differences in the composition of subdominant Symbiodiniaceae may lead to differences in the thermal tolerance of Fungiidae on evolutionary branches. Bacterial diversity and community structure showed that the bacterial community of *C. costulata* (*Cycloseris*), in which the earliest evolutionary divergence occurred, had higher β-diversity and more discrete communities than that of other coral species. In contrast, *L. scabra* (*Lithophyllon*), with a relatively recent divergence, had lower α-diversity and a more stable community structure. Thus, Fungiidae with later evolutionary divergence may be more thermotolerant, whereas the coral species that evolved earlier may be more sensitive to heat environments. This may result from the increase in microbial regulation ability during the evolutionary divergence of Fungiidae, which enhanced the stability of the bacterial community and the thermal adaptability of the coral holobiont. In addition, potentially beneficial bacteria represented by the norank Terasakiellaceae, *Pseudoalteromonas*, *Thermus*, *Alteromonas*, and BD1-7 clade dominated the bacterial community of Fungiidae (relative abundance varied between 29% and 85%). Still, the pathogenic *Vibrio* was stably present in the core bacterial microbiome. Beneficial bacteria may be a potential source of heat tolerance acquisition and a key factor for Fungiidae to be winners during heat stress events, and the stable presence of pathogenic *Vibrio* in the core bacterial microbiome suggests that Fungiidae may be at risk for potential thermal bleaching, both of which together shape the thermal tolerance of Fungiidae. Moreover, complex interactions were observed between the dominant Symbiodiniaceae sub-clades and bacteria. The main mode of interaction in the Fungiidae microbiome was co-occurrence correlation. This indicates that close microbial partnerships may help improve the thermal tolerance of coral holobionts. The microbial interaction network of potential ancestral clade II possessed lower complexity, greater resilience, higher stability, and higher information/energy transfer efficiency, suggesting that it may be more heat-tolerant than potential ancestral clade I. Accordingly, we revealed that evolutionary radiation and microbial community dynamics shaped the thermal tolerance of Fungiidae in the southern SCS, providing new insights for assessing the thermal adaptation of corals in the context of global warming.

## MATERIALS AND METHODS

### Study area and sample collection

SJR (10.17 N, 115.27 E) is located in the southern part of the SCS, a nearly pear-shaped enclosed atoll with a length of about 5.75 km, a width of about 3.25 km, and a total area of about 16 km^2^ (Fig. 1) (142). Through SCUBA diving, 28 Fungiidae samples were collected from seven stations (at depths ranging from 2 to 15 m) in SJR, including *Danafungia horrida*, *Halomitra pileus*, *Lithophyllon scabra*, *Podabacia crustacea*, *Cycloseris costulata*, *Herpolitha limax*, and *Lobactis scutaria*. After the collected samples were transferred to the ship, the surface of coral tissue was cleaned with artificially sterile seawater (salinity: 35‰) to avoid interference of free-living microorganisms in seawater with subsequent molecular experiments. Subsequently, all samples were transferred into 15-mL cryotubes (Jet Biofil, Guangzhou, China), and samples were immediately stored at −20°C until DNA extraction.

### DNA extraction, PCR amplification, Sanger sequencing, and Illumina sequencing

The TIANamp Marine Animals DNA Kit (Tiangen, Beijing, China) was used to extract the DNA of Fungiidae following the manufacturer’s instructions. After quality testing, qualified DNA samples were selected as templates for PCR amplification. The COI gene was amplified using COI F (5′-CTGCTCTTAGTATGCTTGTA-3′) and COI R (5′-TTGCACCCGCTAATACAG-3′). Based on the ABI GeneAmp 9700 thermal cycler, PCR reactions were carried out under the following conditions: 30 s at 94°C, followed by 30 cycles of 94°C for 30 s, 55°C for 45 s, 72°C for 90 s, and a final extension at 72°C for 5 min. After amplification, the PCR products were purified using the QIAquick PCR Purification Kit (QIAGEN, Hilden, Germany), and the purified products were shipped to Sangon Biotech (Shanghai, China) Co., Ltd. for sanger sequencing.

In addition, nucleic acids from the coral microbiome were extracted using the DNeasy Plant Mini Kit (QIAGEN). Qualified DNA samples were used as the templates, and the Symbiodiniaceae rDNA ITS2 gene was amplified using the ITSintfor2 (5′-GATTGCAGAACTCCGTG-3′) and ITS2-reverse (5′-GGGATCCATATGCTTAAGTTCAGCGGGT-3′), and the highly variable V3-V4 region of the 16S rRNA bacterial gene was amplified using the 338F (5′-ACTCCTACGGGAGGCAGCAG-3′) and 806R (5′-GGACTACHVGGGTWTCTAAT-3′). ABI GeneAmp 9700 thermal cycler with the following program: ITS2: 3 min at 94°C, followed by 35 cycles of 94°C for 30 s, 55°C for 30 s, 72°C for 45 s, and a final extension at 72°C for 10 min; 16S rRNA: 3 min at 95°C, followed by 29 cycles of 95°C for 30 s, 53°C for 30 s, 72°C for 45 s, and a final extension at 72°C for 10 min. The PCR products were purified using the QIAquick PCR Purification Kit (QIAGEN) and subsequently sequenced in 2× 300 bp mode using the Illumina Miseq platform at Majorbio Biopharm Technology Co., Ltd. (Shanghai, China).

### Molecular clock analysis

Searching the age range recorded for the fossils of each genus of Fungiidae through http://www.fossilworks.org/cgi-bin/bridge.pl. Based on the sequences obtained by the COI gene in the Materials and Methods, MEGA was used to construct a phylogenetic prior tree file of Fungiidae based on the maximum likelihood method. The calculation of the optimal nucleotide substitution model was performed on PhyloSuite. BEAUTi was used to build the preparation files for the BEAST. Among them were the taxa option for setting the classification subset based on the phylogenetic prior tree; the optimal nucleotide substitution model was set in the sites option; the clock model was set to strict clock; the tree priors were set to yule process; the priors option could perform the time calibration node settings; and the number of generations of the molecular clock was set to 10,000,000. The molecular clock obtained by BEAST was examined on Tracer. If the effective sample size value >200 was considered a valid molecular clock, <200 then increase the number of running generations and reconstruct the molecular clock. TreeAnnotato was used to synthesize and obtain the tree files, and FigTree was used to view the species divergence time.

### Bioinformatics processing and statistical analyses

Microbiome sequence processing was performed using the Quantitative Insights Into Microbial Ecology 2 (QIIME 2) framework (143). Strict quality control and filtering procedures were applied to the ITS2 and 16SrRNA genes generated by the Illumina MiSeq platform. Specifically, the DADA2 pipeline eliminated low-quality reads and chimeric sequences from Symbiodiniaceae and bacteria. Subsequently, the qualified sequences were flattened based on the minimum sample requirement, and the Symbiodiniaceae and bacterial reads were clustered into an ASV using the DADA2 algorithm. It is important to note that the presence of multicopy, intragenomic variation, and pseudogenes can overestimate Symbiodiniaceae abundance and diversity (144–146). To enhance the accuracy of the results, it is recommended to combine quality-controlled ITS2 sequences with ITS2 sequence comparisons and ASV analyses. In the ITS2 sequence comparison analysis, quality-controlled ITS2 sequences were compared to the ITS2 database of Symbiodiniaceae using local BLASTn, and the specific comparison scheme and code can be found here (34). In this study, ITS2 sequences with a relative abundance exceeding 5% in at least one sample were set to meet the quality control requirements of dominant Symbiodiniaceae sub-clades (147). For ASV analysis, ASV representative sequences were aligned to the ITS2 database using BLASTn to remove non-Symbiodiniaceae ASVs (34). Additionally, we compared the ASV representative bacterial sequences to the SILVA 132 database using QIIME 2’s feature classifier (143, 148), with a confidence threshold of 0.7 (150). During the comparison, we excluded ASVs belonging to chloroplasts, mitochondria, and other non-bacteria to obtain species annotation information for the 16S rRNA gene.

The diversity indices of Symbiodiniaceae and bacteria at the ASV level, including the Chao, Shannon diversity, and Simpson evenness indices, were calculated using Mothur. Based on R, the Kruskal-Wallis test was used to determine the differences between groups in the diversity indices (significance level threshold of 0.05), and a post hoc test was performed using the Student-Newman-Keuls test. PCoA was performed using the Vegan package in R based on the Bray-Curtis distance algorithm, which can analyze the diversity of bacteria and reveal variability in the community structures of Symbiodiniaceae and bacteria. This was combined with PERMANOVA (9,999 permutations) to test the significance of the differences in the community structures of Symbiodiniaceae and bacteria. ASV types of bacteria with different enrichment among coral species were tested using LEfSe (LDA = 2.0; *P* < 0.05). Additionally, QIIME 2 was used to identify the core bacterial microbiome (149). The Vegan package was used to visualize the heat map of the core microbiome bacteria at the ASV level.

### Microbial interaction network analysis

Statistical results based on the relative abundance of dominant Symbiodiniaceae sub-clades and bacterial ASVs in the Materials and Methods and the CoNet plugin in Cytoscape 3.9.1 were used to construct the microbial interaction network of Fungiidae (124, 150). Pearson and Spearman correlations were used to detect correlations between Symbiodiniaceae and bacteria, and Bary-Curtis and Kullback-Leibler distances were used to detect heterogeneity between Symbiodiniaceae and bacteria. Then 1,000 positive and 1,000 negative edges were set as thresholds for the microbial interaction network relationships for randomized network recovery. The significance *P*-values of microbial interactions were merged and calculated using Brown’s method (151), and multiple comparisons were corrected using the Benjamini-Hochberg procedure (152). Results with significance <0.05 after the combined calculation were used for downstream analysis. The visualization programs Gephi and Cytoscape were used to recover and map the microbial interaction networks, and only co-occurrence correlations are shown in the figure. The complexity of the interaction network was calculated as the ratio of the total number of edges to the total number of nodes in the network (54). In addition, the topological properties of the microbial interaction networks were calculated using the igraph package in R, including network diameter, network centralization, average path length, average degree, clustering coefficient, modularity, and betweenness centrality. The network diameter describes the scale of the network (138). The average path length is closely related to information or energy transmission efficiency within a network (137). The average degree is defined as the average number of interactions per node, which reflects the strength of interactions among microbial taxa (153). The clustering coefficient measures the degree to which network nodes tend to cluster, indicating the stability and robustness of the microbial interaction network structure (138, 139). The betweenness centrality can reflect the resilience and connectivity of microbial communities (132, 135).

## Data Availability

The Sanger raw data have been submitted to GenBank (accession numbers OR077862-OR077889). The NGS raw read data set of these two amplicons has been submitted to the NCBI (accession numbers PRJNA978957 and PRJNA978948).

## SUPPLEMENTAL MATERIAL

Table S1 spectrum.02436-23-s0001.xlsx Monthly average SST (2012-2022).

Table S2 spectrum.02436-23-s0002.xlsx The relative abundance of beneficial and pathogenic bacteria.

Table S3 spectrum.02436-23-s0003.xlsx Core bacterial microbiome of Fungiidae.

ASM does not own the copyrights to Supplemental Material that may be linked to, or accessed through, an article. The authors have granted ASM a non-exclusive, world-wide license to publish the Supplemental Material files. Please contact the corresponding author directly for reuse.
